# Metabolic Profiling of Intact *Arabidopsis thaliana* Leaves during Circadian Cycle Using ^1^H High Resolution Magic Angle Spinning NMR

**DOI:** 10.1371/journal.pone.0163258

**Published:** 2016-09-23

**Authors:** D. Augustijn, U. Roy, R. van Schadewijk, H. J. M. de Groot, A. Alia

**Affiliations:** 1 Leiden Institute of Chemistry, POB 9502, 2300, RA, Leiden, The Netherlands; 2 Institute of Medical Physics and Biophysics, University of Leipzig, Härtelstr. 16–18, D-04107, Leipzig, Germany; National Research Council of Italy, ITALY

## Abstract

*Arabidopsis thaliana* is the most widely used model organism for research in plant biology. While significant advances in understanding plant growth and development have been made by focusing on the molecular genetics of *Arabidopsis*, extracting and understanding the functional framework of metabolism is challenging, both from a technical perspective due to losses and modification during extraction of metabolites from the leaves, and from the biological perspective, due to random variation obscuring how well the function is performed. The purpose of this work is to establish the *in vivo* metabolic profile directly from the *Arabidopsis thaliana* leaves without metabolite extraction, to reduce the complexity of the results by multivariate analysis, and to unravel the mitigation of cellular complexity by predominant functional periodicity. To achieve this, we use the circadian cycle that strongly influences metabolic and physiological processes and exerts control over the photosynthetic machinery. High resolution-magic angle spinning nuclear magnetic resonance (HR-MAS NMR) was applied to obtain the metabolic profile directly from intact *Arabidopsis* leaves. Combining one- and two-dimensional ^1^H HR-MAS NMR allowed the identification of several metabolites including sugars and amino acids in intact leaves. Multivariate analysis on HR-MAS NMR spectra of leaves throughout the circadian cycle revealed modules of primary metabolites with significant and consistent variations of their molecular components at different time points of the circadian cycle. Since robust photosynthetic performance in plants relies on the functional periodicity of the circadian rhythm, our results show that HR-MAS NMR promises to be an important non-invasive method that can be used for metabolomics of the *Arabidopsis thaliana* mutants with altered physiology and photosynthetic efficiency.

## Introduction

As a model organism, *Arabidopsis thaliana* play a central role in understanding biological functions across plant species and in characterizing phenotypes associated with genetic mutations [1]. Significant advances in understanding plant growth and development have been made by focusing on molecular genetics of *Arabidopsis*. Several high-throughput technologies to produce information of the transcriptome, metabolome, proteome, interactome, and other omics data sets are available [2,3]. However, understanding the functional framework of metabolism in native state in leaves poses a major challenge for all metabolomics approaches. Many approaches, including mass spectrometry as well as NMR methods require labour-intensive extraction of plant metabolites which can cause biases resulting from differential extraction efficiencies and from the loss of volatile metabolites [4,5]. Extraction methods also cause the loss of molecular information regarding specific associations within and between polymeric structural plant components.

Understanding the functional framework of metabolism is also challenging from biological perspective due to random variations obscuring how well the function is performed. It has been argued that periodicity in biological system such as circadian rhythms can provide robustness that helps to tolerate the random variations [6]. Many biological systems rely on functional periodicity, as is evidenced by abnormal or chaotic behaviour when functional periodicity is lost. In plants, the circadian cycle strongly influences metabolic and physiological processes [7–10]. The endogenous biological clock allows plants to anticipate on daily changes in the environment, such as the onset of dawn and dusk, providing them with an adaptive advantage. It has been shown that growth, productivity and competitive advantage in plants are enhanced by matching the circadian cycle with the external light/dark cycle [7]. The internal clock also regulates physiological processes, including photoperiodic induction of flowering, hypocotyl elongation, cotyledon movement and stomatal opening [8–10]. Previous studies reported large diurnal changes in the expression of several genes in *Arabidopsis* [11,12]. Diurnal changes of few soluble metabolites have also been reported in extracts of *Arabidopsis* leaves [13]. The current understanding of diurnal changes in metabolites has been based on destructive analysis of individual components [11–13]. These in vitro results may not faithfully reflect the native structural and conformational information. Examining the rhythmic pattern of metabolites directly in the intact *Arabidopsis* leaves without any extraction during circadian cycle would be important to understand the functional framework of metabolism in native state.

High resolution magic angle spinning (HR-MAS) NMR offers fast and sensitive method to study molecules in intact samples *in situ* and *in vivo*. HR-MAS NMR is viewed as a hybrid technique between solution state NMR and solid state NMR. Similar to solid state NMR, the use of magic angle spinning (MAS) effectively removes spectral line broadening resulting from magnetic susceptibility, homonuclear dipolar interactions and chemical shift anisotropy. When the sample is spinning along the magic angle of θ = 54.7° with respect to the static magnetic field (B_0_), line broadening effects are reduced to zero because the (3 cos^2^θ—1)/2 part of the Hamiltonian disappears [14]. Thus, HR-MAS NMR yields narrow lines in heterogeneous samples such as tissue or whole cells. At the same time it retains the advantages of low power levels and deuterium locking in classical solution NMR experiments for obtaining good stability, resolution and overall performance of the NMR experiment. Similar to solution NMR, HR-MAS NMR involves direct polarization transfer and not cross polarization transfer (CPMAS) between ^1^H and other nuclei (^13^C or ^15^N), thus differentiating it from CPMAS experiments on true solids. The application of HR-MAS NMR has been earlier reported for studying chemotype variations in frozen leaf and root samples of *Withania somnifera* [15], to monitor alterations in metabolite profile of *Jatropha curcas* during virus infection [16] and to detect metabolites in extracts from *Arabidopsis* [17]. In addition, the application of HR-MAS NMR for metabolite monitoring has been reported for Italian sweet pepper [18], Italian garlic [19], citrumelo [20], and for tree species such as poplar [21] and *Euglena* [22] (see S1 Table). To our knowledge HR-MAS NMR was not yet applied to intact fresh leaves of *Arabidopsis thaliana*.

The objective of the present study was to establish the metabolic profile directly from the *Arabidopsis thaliana* leaves without metabolite extraction using HR-MAS NMR spectroscopy and to study functional framework of metabolism by following metabolic rhythm throughout the light/dark cycle. Our results demonstrate that HR-MAS NMR on intact *Arabidopsis* leaves represents a novel platform that could provide important *in vivo* information of regular metabolic network against which altered metabolic profile due to stress, infection or mutation can be assessed.

## Materials and Methods

### Material

Seeds of wild-type *Arabidopsis thaliana* plants of ecotype Colombia O were incubated in petri dishes on wet filtration paper and transferred to small tubes filled with soil and sand mixture (Holland Potgrond). To synchronise germination, the tubes were kept in complete darkness at 277 K for 72 hours. Germinated seeds were transferred to the greenhouse and maintained at 293 K under a 12 hours light (100 μmol m^-2^s^-1^) and 12 hours dark regime for 4 weeks, at which time flowering had not commenced.

### Sample collection and preparation for NMR analysis

**Fig 1:**
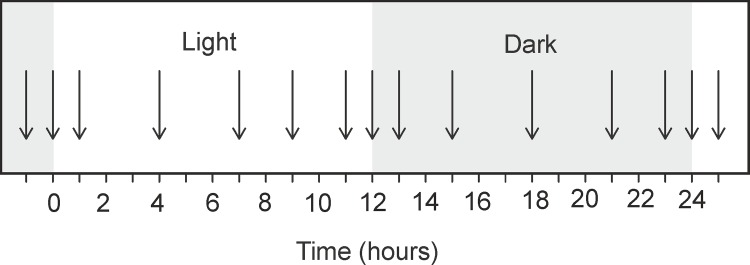
Time points of harvesting of *Arabidopsis* leaves during circadian cycle. Rosette leaves were collected at 15 time points from non-flowering *Arabidopsis* plants during growth stage 3.70–3.90.

Typically, 8–10 replicate samples of intact rosette leaves were collected at 15 time points during the entire photoperiod (Fig 1). For observing the internal metabolite rhythm during free running conditions, the plants were transferred to continuous dark for 48 hours. Intact rosette leaves were then collected at 15 time points within 24 hours. A single leaf was rolled and inserted into a 4mm Zirconium Oxide rotor. 10 μl of deuterated phosphate buffer (100 mM, pH 6) containing 0.1% (w/v) 3-trimetylsilyl-2,2,3,3-tetradeuteropropionic acid (TSP) was added as a lock solvent and NMR reference, respectively. The rotor was placed immediately inside the NMR spectrometer. For each time point 8 replicates were measured.

### ^1^H high resolution magic angle spinning (HR-MAS) NMR spectroscopy

All experiments were carried out with a Bruker DMX 400 MHz NMR spectrometer operating at a proton resonance frequency of 399.427 MHz and equipped with a 4 mm HR-MAS dual inverse ^1^H/^13^C probe with a magic angle gradient. Data were collected with a spinning rate of 4 kHz. A temperature of 277 K was used to avoid any tissue degradation during data acquisition. The temperature was stabilized with a Bruker BVT3000 control unit.

One-dimensional ^1^H HR-MAS NMR spectra were recorded using the rotor synchronized Carr-Purcell-Meiboom-Gill (CPMG) pulse sequence with water suppression (for details see S1 Fig [23]. Each one-dimensional spectrum was acquired in 16k data points applying a spectral width of 8000 Hz. The number of averages was 256, with 8 dummy scans. A constant receiver gain of 1024, an acquisition time of 2 seconds and a relaxation delay of 2 seconds were used. Since NMR measurements were done on intact tissue, the relaxation delay was set to a small value to remove nascent short T_2_ components due to presence of lipids. Spectra were processed by applying an exponential window function corresponding to a line broadening of 1 Hz and data were zero-filled prior to Fourier transformation. ^1^H HR-MAS NMR spectra of plant tissue were phased manually and automatically baseline corrected using TOPSPIN 2.1 (Bruker Analytische Messtechnik, Germany).

To confirm the assignments, two-dimensional homonuclear correlation spectroscopy (^1^H-^1^H COSY) was performed using Bruker’s standard pulse program library. The parameters used for COSY were: 2048 data points were collected in the t_2_ domain over the spectral width of 4k Hz, 512 t_1_ increments were collected with 64 transients, relaxation delay 1 sec, acquisition time 116 msec. The data were zero-filled to 512 data points and were weighted with a sine bell window function in both dimensions prior to Fourier transformation. 2D J-resolved spectrum was measured using pulse sequence (“jresqfpr”), from the Bruker pulse program library. Representative ^1^H-J-resolved spectra is shown in S2 Fig.

### Quantification of metabolites

NMR data analysis was performed using MestReNova software version 10.0.1–14719 (Mestrelab Research S.L. Spain). The concentrations of the various metabolites in the spectra of intact leaf were determined by comparing the integral peak intensity of the metabolite of interest with that of the TSP peak, after correcting for the number of contributing protons and for tissue weight. All statistical analysis (t-tests and ANOVAs) of the NMR quantification results were performed with OriginPro v. 9 (Northampton, USA). F-values were calculated, and F-values larger than 2.8 (p<0.05) were considered significant.

### Multivariate statistical analysis

Multivariate statistical analysis of primary metabolites in the spectra was performed using the Bruker software package AMIX (version 3.8.6). The one-dimensional CPMG spectra, collected from leaf samples at 1 h, 7 h, 12 h and 23 h (see Fig 1), were subdivided in the range between 0.3 and 9 ppm into buckets of 0.04 ppm (total 218 buckets), using Bruker AMIX software (Versio 3.8.7, Bruker GmbH). The region of 4.20–6.00 ppm was excluded from the analysis to remove the water signal. To compensate for the differences in the overall metabolite concentration between individual samples, the data obtained were mean centered, scaled to unit variance and then normalized by dividing each integral of the segment by the total area of the spectrum [24]. The resulting data matrix was exported into Microsoft office Excel (Microsoft Corporation, USA). This was then further imported into SIMCA software (Umetrics AB) for multivariate statistical analysis.

## Results and Discussion

### Identification of metabolites

**Fig 2:**
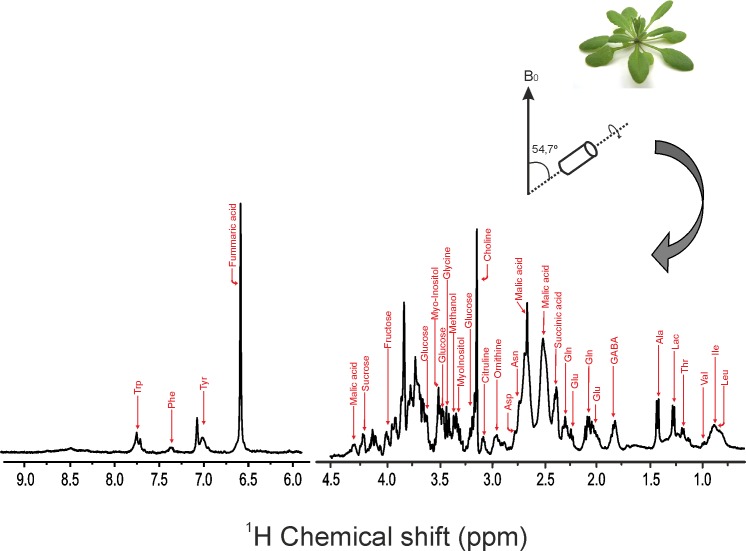
Metabolic profile of intact *Arabidopsis* leaf. A representative one-dimensional ^1^H HR-MAS NMR spectrum, obtained from intact leaf of *Arabidopsis thaliana* at t = 7 hours, showing resonance assignment of several metabolites (see Table 1 for assignement). Abbreviation: Leucine (Leu); Isoleucine (Ile); Valine (Val); Threonine (Thr); Lactic acid; Alanine (Ala); GABA; Glutamate (Glu); Glutamine (Gln); Asparagine (Asn); Aspartic acid (Asp); Glycine (Gly); Tyrosine (Tyr); Phenylalanine (Phe); Tryptophane (Trp).

**Fig 3:**
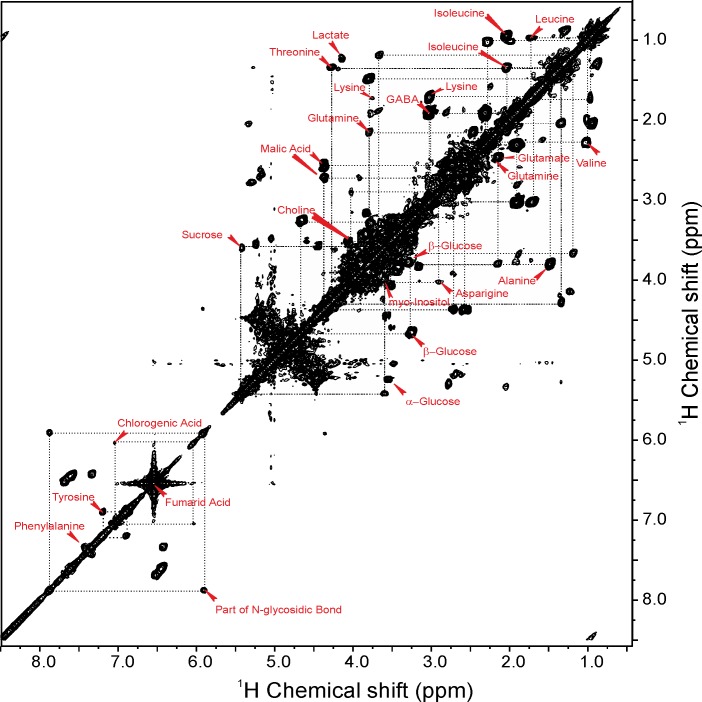
Two-dimensional HR-MAS spectra of intact *Arabidopsis* leaf. A representative two-dimensional HR-MAS ^1^H-^1^H COSY spectrum obtained from intact leaf of *Arabidopsis thaliana* measured at 400 MHz DMX NMR spectrometer (Bruker, Germany) at a spinning speed of 4 kHz. The ^1^H shifts were calibrated using TSP as an internal standard.

Since the metabolites in intact cells of leaves may differ dramatically in their abundance, size, location and relative mobility, a rotor-synchronized Carr–Purcell–Meiboom–Gill (CPMG) pulse sequence coupled with water suppression was used to improve sensitivity and to generate a better baseline by removing backgrounds, resulting from superimposition of molecules in low abundance and/or with restricted mobility. A representative one-dimensional ^1^H HR-MAS NMR spectrum obtained directly from intact leaf of *Arabidopsis thaliana* at t = 7 hours is shown in Fig 2. Peak assignment was performed according to earlier literature and the Biological Magnetic Resonance Data Bank (BMRB) [25,26]. A list of identified metabolites is given in Table 1. The ^1^H HR-MAS NMR spectrum could be divided into three major regions. The high-field region (0.0–3.0 ppm) was rich in amino acids, the mid-field region (3.0–5.5 ppm) contained sugars and the down-field region (5.5–10.0 ppm) was dominated by aromatic compounds. In the high-field region, several metabolites were identified including alanine, threonine, lactic acid, γ-aminobutyric acid (GABA), glutamate and malic acid. Signals from mid-field region showed diverse sugars including glucose, fructose and sucrose. Signals from fumaric acid, tyrosine, tryprophane and phenylalanine were observed in the down-field region. The results were corroborated by two-dimensional COSY spectra (Fig 3) and *J-*resolved spectra (S2 Fig) to resolve complexity of overlapping and interfering spectral regions to allow for correct identification of metabolites. Detailed assignment of sugar region in two-dimensional COSY spectrum is shown in S3 Fig and S4 Fig.

**Table 1:** ^1^H Chemical shift assignment of metabolites in leaves of wild-type *Arabidopsis Thaliana*.

Compound	Assignment [25,26]	Chemical shift (ppm)	Multiplicity [25,26]	*Connectivity*
Alanine (Ala)	^2^CH	3.76	q	2–3
	^3^CH_3_	1.46	d	
Aspartic Acid (Asp)	^2^CH	3.90	dd	2–3
	^3^CH_2_	2.80	dd	
Asparagine (Asn)	^2^CH	4.00	dd	2–3
	^3^CH_2_	2.80	m	
Choline	^1^CH_2_	4.05	m	1–2
	^2^CH_2_	3.50	m	
	N(CH_3_)_3_	3.22	s	
Citruline	^5^CH_2_	3.13	t	
Fructose	^3^CH	4.03	t	4–5
	^4^CH	3.89	dd	
Fumaric Acid	CH = CH	6.60	s	
GABA	^4^CH_2_	2.28	t	4–3
	^3^CH_2_	1.89	m	3–2
	^2^CH_2_	3.00	t	
α-_D_-glucose (Glc)	^1^CH	5.22	d	1–2
	^2^CH	3.52	dd	2–3
	^3^CH	3.70	t	
β-_D_-glucose (Glc)	^1^CH	4.63	d	1–2
	^2^CH	3.230	dd	2–3
	^3^CH	3.47	t	
Glutamate (Glu)	^2^CH	3.74	dd	
	^3^CH_2_	2.12	m	2–3
	^4^CH_2_	2.35	m	3–4
Glutamine (Gln)	^3^CH_2_	2.11	m	3–4
	^4^CH_2_	2.43	m	
Glycine	^2^CH_2_	3.54	s	
Isoleucine (Ile)	^3^CH_3_	1.968	m	3–5
	^4^CH_2_	1.248	m	3–6
	^5^CH_3_	0.92	t	
	^6^CH_3_	0.98	d	
Lactic acid	^2^CH	4.10	q	2–3
	^3^CH_3_	1.32	d	
Leucine (Leu)	^4^CH	1.7	m	4–5
	^5^CH_3_	0.948	t	
Malic Acid	^2^CH	4.35	dd	
	^5^CH_2_	2.64	dd	2–5
	^5`^CH_2_	2.50	dd	2–5`
Methanol	CH_3_	3.35	s	
Myo-Inositol	^1^CH	3.52	dd	1–2
	^2^CH	4.05	t	
Ornithine	^5^CH_2_	3.02	t	
Phenylalanine (Phe)	^4^CH	7.36	m	4–6
	^6^CH	7.32	m	
Succinic Acid	^2^CH_2_,	2.39	s	
	^3^CH_2_	2.39	s	
Sucrose	^3^CH	4.22	d	
	^7^CH	5.42	d	7–12
	^12^CH	3.59	dd	
Threonine (Thr)	^3^CH	4.24	m	3–4
	^4^CH_3_	1.32	d	
Tryptophan (Trp)	^4^CH	7.73	d	
Tyrosine (Tyr)	^2^CH	7.19	m	2–3
	^3^CH	6.89	m	
Valine (Val)	^3^CH_2_	2.261	m	3–4
	^4^CH_3_	1.029	d	

Among several metabolite signals, twelve primary metabolites were quantified by integrating the distinct characteristic signals of each metabolite with respect to the intensity of the nine protons of TSP on the fresh weight basis. These metabolites include organic acids (fumaric acid, malic acid and lactic acid), sugars (glucose, fructose), amino acids (glutamate, alanine, phenylalanine, tyrosine, aspartic acid and γ-aminobutyric acid (GABA)), and precursor of membrane phospholipids (e.g. choline). The concentration of these primary metabolites at t = 7 hours are shown in Table 2.

**Table 2:** Metabolite levels in leave of *Arabidopsis thaliana* at t = 7 hours. Results are mean ± standard error.

Metabolite	Concentration (mg/g FW)
Fumaric acid	4.276 ± 0.566
Malic acid	23.13 ± 5.536
Lactic acid	1.308 ± 0.234
Glucose (α-anomer + β-anomer)	12.52 ± 1.938
Fructose	1.412 ± 0.209
Glutamate	2.885 ± 0.762
Alanine	8.984 ± 0.821
Phenylalanine	0.009 ± 0.003
Tyrosine	4.590 ± 0.361
Aspartic acid	2.972 ± 0.593
GABA	10.80 ± 2.306
Choline	1.510 ± 0.082

### Characterization of metabolites throughout the circadian cycle

**Fig 4:**
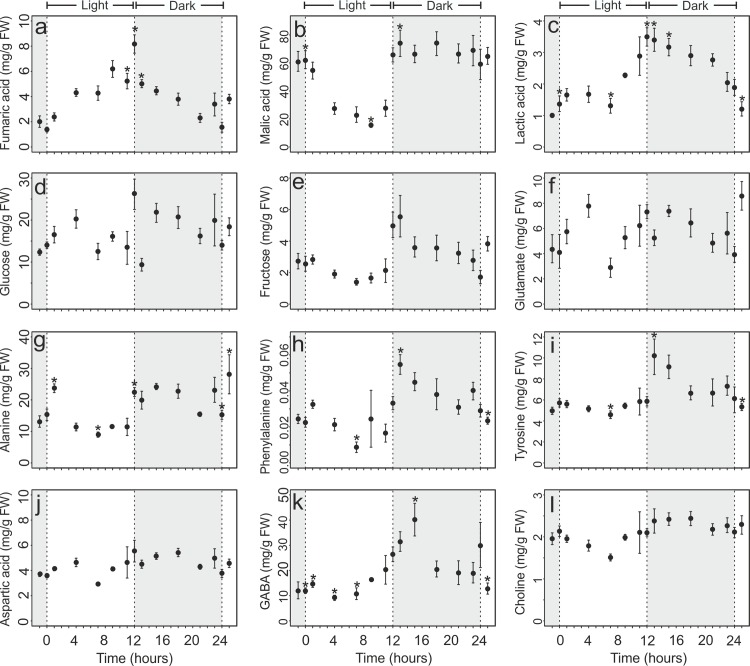
Changes in the levels of metabolites during circadian cycle in leaves of *Arabidopsis thaliana*. Wild-type *Arabidopsis thaliana* (Col-0) were grown in a 12-h-light/12-h-dark cycle as described in method section. The whole intact rosette leaf was harvested at different time points (as shown in Fig 1) during light and dark period. The results are given as the mean of 8 replicates ± standard error (* p < 0.05, **p < 0.01).

The circadian cycle strongly influences many plant metabolic and physiological processes [7–10]. Previous studies reported large diurnal changes in the expression of many genes in *Arabidopsis* [11–13]. To understand the functional framework of metabolism in native state in leaves during circadian cycle, we examined metabolites at different time points of the circadian cycle directly in the intact leaves. The stacked plots at different time points is shown in (S5 Fig). Fig 4 show significant and consistent rhythmic pattern of several metabolites during circadian cycle.

**Fig 5:**
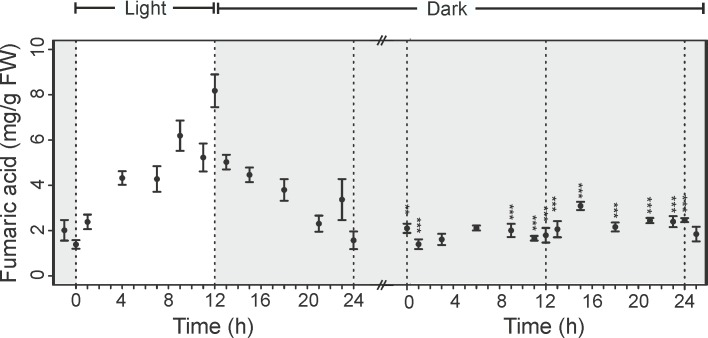
Changes in the levels of fumaric acid in the leaves of *Arabidopsis thaliana*. Fumaric acid was measured in intact leaves at different time points during: 12h light/12h dark period (A) and during continuous dark period (B). Results are mean of 4 replicates ± standard error (** p < 0.01, *** p < 0.001).

Fumaric acid participate in a multiplicity of pathways in plant metabolism, however its function as carbon stores in C3 plants has not been deeply addressed. While in C3 plants, the major photoassimilates are starch and soluble sugars, in some of the C3 plants, including *Arabidopsis*, fumaric acid is considered to be one of the major form of fixed carbon [27]. Previous studies have indicated that similar to starch and soluble sugars, fumerate can be metabolized to yield energy and carbon skeletons for production of other compounds. Fig 4A shows that fumaric acid concentrations increases during the day, reaching maximum at the end of light period and then started decreasing and reached its minimum level at the end of the dark period. This observation is consistent with previous study showing high level of fumaric acid during light period measured in the extract of *Arabidopsis* leaves by GC-MS [28]. Interestingly, the concentration of fumaric acid dropped to a steady level in *Arabidopsis* shifted to extended dark (Fig 5B). A possible explanation is that the formation and the degradation rate of fumaric acid may be equal during continuous dark. It is also possible that fumaric acid is transported out of the leaves during growth in continuous dark [27].

Malic acid is another carbon storage molecule which participate in various pathways in plant metabolism and also plays an important role in CAM and C4 photosynthesis [28]. Malic acid concentration showed a decreasing pattern during the light period, while it increased during dark period and remained high during the dark period (Fig 4B). This is in contrast to earlier studies where diurnal malic acid changes assayed by GC-MS in *Arabidopsis* grown in a 16h light/8h night regime showed a high level of malic acid during end of day and declined during night time [29]. This difference could be attributed to differences in light/dark regime [13] used in previous study as well as extraction methods used which cause the release of malic acid from different compartments in the plant cells.

Malic acid is dominantly compartmentalised in vacuole [30,31]. In our study, the signals of ^5´^CH_2_ and ^2^CH of malic acid were slightly shifted (observed at 2.5 ppm and 4.35 ppm, respectively) as compared to the signals of malic acid in water at pH 7.0 (^5`^CH_2_ and ^2^CH at 2.35 and 4.29, respectively) [32]. Similar shifts of resonances of malic acid has been observed in leaves in earlier HR-MAS NMR studies [15], which may be due to the influence of native acidic environment of the vacuole, where excess of malic acid is stored. Isolating Malic acid by extraction methods not only remove the compartmentalised information, but also may cause potential chemical changes or degradation due to absence of native environment. Loss of compartmental information may mask critical details on its ability to perform *in vivo* rhythmic function. Thus non-invasive measurement of Malic acid by HR-MAS NMR as shown in this study has clear advantage in monitoring diurnal changes in malic acid in its native environment.

Fig 4C shows rhythmic changes in lactic acid concentration. The concentration of lactic acid was highest at the end of the light period and dropped during the dark period. The role of lactic acid in *Arabidopsis* leaves is still unclear and the diurnal changes in lactic acid have not been studied so far. However, it has been recognised in previous studies that insertion of electrons coming from lactic acid into the respiratory electron transport chain in light dependent [33].

The sugar derivatives, glucose and fructose levels are reported to be influenced by light/dark period [11,12]. (Fig 4D and 4E) show the concentration of glucose and fructose throughout the light/dark cycle. An increasing trend was observed in the glucose concentration during the light period, which declined during the dark period. The concentration of fructose in the leaves first declined during the light period followed by a rise to a maximum amount at the end of the light period. Subsequently it declined during the dark period. During the light period, photosynthetic carbon fixation takes place in the leaves and photosynthate is stored primarily in the form of sugars. These sugars are then utilized during the dark period [11]. The circadian rhythm of sugars are known as an important player in the global regulation of diurnal gene expression [11,34,35]. The low concentration of sugars at the beginning of the light period ensures the repression of the *PSEUDO-RESPONSE REGULATOR7* (*PRR7*) promoter, leading to the activation of the morning-expressed *CIRCADIAN CLOCK ASSOCIATED1* (*CCA1*) and LATE ELONGATED HYPOCOTYL (LHY) genes in the central loop [34,35].

The glutamate level was elevated during the light period and dropped during the dark period as shown in Fig 4F. The glutamate level is known to be dependent on available nitrate [36]. Nitrate is taken up from the soil and is converted to ammonium in the leaves or roots and reduced through the glutamine synthetase/glutamate synthase (GS/GOGAT) pathway, to glutamate or glutamine [36,37]. Recent studies have shown that nitrate assimilation is stimulated by light [11], which may be responsible for the elevation of glutamate as seen in the present study (Fig 4F). The α-amino group of glutamate can be transferred to other amino acids via various aminotransferases [37]. Thus the rhythmic pattern of glutamate may influence the level of other amino acids throughout the light dark cycle. (Fig 4G–4J) illustrates that alanine, phenylalanine, tyrosine as well as aspartic acid show a clear rhythmic pattern during light dark cycle. Glutamate is also known to be converted into the ubiquitous non-protein amino acid γ-aminobutyric acid (GABA), an import player in plant carbon metabolism, mainly because it can bypass two steps in the TCA cycle [38,39]. This is especially useful when the plants are grown under carbon-limited conditions. Fig 4K show that GABA concentration in *Arabidopsis thaliana* leaves was enhanced during light period and decreased during later in the dark period. This phenomenon was also observed in an earlier study of Fahnenstich et al using GC-MS analysis [29].

The levels of choline has also been monitored during light dark period (Fig 4L). The concentration of choline decreased during light period and increased during the dark period (Fig 4L). Choline is an essential metabolite in plants needed to synthesize membrane phospholipids. It is widely distributed and occurs in relatively high concentrations in many plant tissues as free choline, lipid choline (phosphatidyl choline), and sometime as water-soluble bound choline (phosphocholine and glycerophosphocholine) [40,41]. Up to now, the pattern of choline during a light/dark period has not been studied in *Arabidopsis*. It is known, however, that the time of the day influences the membrane lipid composition of *Arabidopsis thaliana* leaves [42,43]. Our results indicate that periodic behaviour of choline may be closely linked with periodic changes in membrane lipids composition.

### Multivariate analysis of ^1^H HR-MAS NMR spectra of *Arabidopsis thaliana* leaves

**Fig 6:**
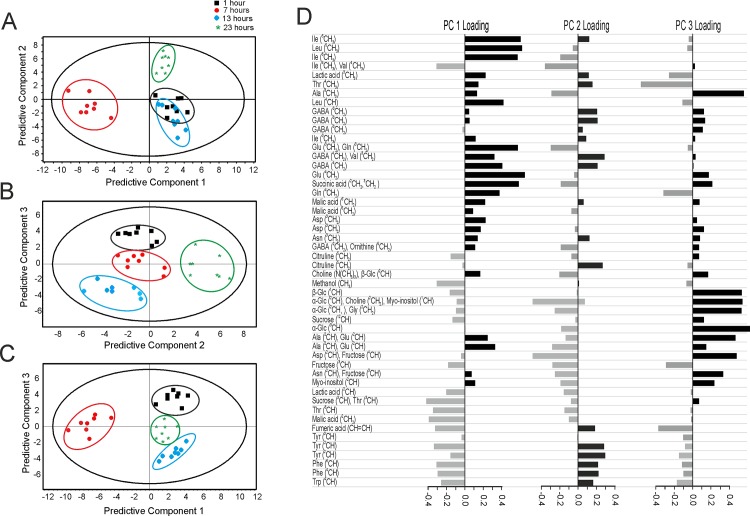
Orthogonal partial least squares discriminant analysis (OPLS-DA) of samples collected at different times during the circadian cycle from wild-type *Arabidopsis thaliana*. The data set contains 8 replicate samples of each 4 time points (1h, 7h into light and13h, 24h into the dark period) in the circadian cycle. (A-C) score plots of OPLS-DA analysis of 1D HR-MAS NMR spectra showing the variable responsible for the separation among samples measured 1 (■), 7 (●), 13 (♦) and 24 (★) hours during circadian cycle. Predictive component 1, 2 and 3 explained 70% of the total variance. (D) Loading plots of predictive component (PC) 1, 2 and 3 for all buckets containing assigned peaks (Table 1). black, positive scores; Gray, negative scores. The metabolite on the vertical axis are arranged in accordance to their chemical shift order in the spectrum (ppm) (see Table 1).

Multivariate analysis (MVA) has proven to be useful in metabolomics. It can explain the variance within a dataset, helps with identification of biologically relevant spectral features or to identify outliers [44]. The two most popular methods for multivariate analysis are unsupervised principal component analysis (PCA) and supervised orthogonal partial least squares discriminant analysis (OPLS-DA). These methods give both a score matrix and a loading matrix, with score matrix showing the relation between observations, while the loading matrix gives the individual contribution of each parameter, which is a peak in the case of NMR spectra [44–48]. Orthogonal partial least squares discriminant analysis (OPLS-DA) was used on the ^1^H HR-MAS NMR spectra from the leaves collected at the beginning of the light period (1 hour), at the middle of the light period (7 hours), at the start of the dark period (13 hours) and at the end of the dark period (23 hours) for covering important time period range. A three-component model explained 100% of the total variance, concluding that the first three predictive components leads to complete separation between the samples collected at four different time points. With the predictive components 1, 2 and 3, 74% of the total variance can be explained (Fig 6A–6C). Even though not completely separated, a clear clustering could be observed from the four time points.

The loading plot of all buckets containing peaks which have been assigned in Table 1 is shown in Fig 6D. Examination of the loadings coming from predictive component 1 show that the separation between the time points arises due to positive loading of GABA, asparate, malic acid, glutamine, glutamic acid, alanine and negative loading corresponding to phenylalanine, tyrosine, fumaric acid and lactic acid. The PC2 loading showed positive loading of phenylalanine, tyrosine, glucose, GABA, aspartate, malic acid, alanine and negative loading of fumaric acid, lactic acid, malic acid, glutamine and glutamic acid. The PC3 loading showed positive loading of glutamine, glutamic acid, glucose, GABA and alanine and negative loading of phenylalnine, tyrosin, fumaric acid and lactate (Fig 6D). This suggested that these metabolites differ in concentration throughout the light/dark cycle. This is also supported by the quantification data shown in Fig 4.

## Conclusion

*In vivo* analysis of metabolic profile during circadian cycle, especially in intact leaves, is an unconquered frontier. The current understanding of metabolic profile in *Arabidopsis thaliana* has been based on analysis with destructive extraction procedures. The analytical methods that provide *in vivo* information could yield important and novel insights into cellular complexity of metabolic pathways in plants. Multidimensional High Resolution Magic Angle Spinning NMR (HR-MAS NMR) has evolved to be a powerful technique in a variety of *in vivo* studies including intact cells. In this study, multidimensional HR-MAS NMR was successfully applied for the first time on intact *Arabidopsis thaliana* leaves to obtain the metabolic profile throughout circadian cycle to unravel cellular complexity predominated by functional periodicity. Multivariate analysis revealed clear variations in primary metabolites at different time points of the light/dark cycle. Knowledge of *in vivo* metabolic profile at different time points in circadian cycle will be important for choosing the particular time point to compare the metabolites pattern in wild-type and different available mutants of *Arabidopsis thaliana* with altered physiology and metabolism.

## Supporting Information

S1 Fig CPMG Pulse sequences used for 1D HR-MAS NMR. D20 = fixed echo time to allow elimination of J modulation effects. D12 = delay for power switching (20μs). L4 = loop for T2 filter.
(TIF)

S2 Fig **HR-MAS** ^**1**^**H-J-resolved spectra of intact *Arabidopsis thaliana* leaf zoomed between spectral region (A) 0.9–2.0 ppm and (B) 3.0 and 4.0 ppm showing assignment of Isoleucine, Leucine, valine (A) and glucose (B).** The *J-*Res spectra were measured using pulse sequence (Jresqfpr) from Bruker’s standard pulse program library. Reference spectra of Isoleucine, Leucine, valine and glucose obtained from Ludwig et al. [Ludwig C, Easton JM, Lodi A, Tiziani S, Manzoor SE, Southam AD, et al. Metabolomics. 2012;8: 8–18] have been overlaid for assignment purpose.
(TIF)

S3 Fig ^1^H-^1^H COSY spectrum of intact *Arabidopsis thaliana* leaf zoomed between 3 and 5.5 ppm region showing assignment of glucose, sucrose and fructose. Reference spectra of glucose (purple), sucrose (green) and fructose (red) [Ulrich EL, Akutsu H, Doreleijers JF, Harano Y, Ioannidis YE, Lin J, et al. Nucleic Acids Res. 2007;36: D402–D408] have been overlaid for assignment purpose.
(TIF)

S4 Fig ^1^H-^1^H COSY spectrum of intact *Arabidopsis thaliana* leaf zoomed between 3 and 5.5 ppm region showing assignment of α-glucose and β-glucose. Reference spectra of **α**-glucose and β-glucose has been overlaid on experimental data with purple colour.
(TIF)

S5 Fig Stacked plots of 1D HR-MAS NMR spectra from intact *Arabidopsis thaliana* leaves. Leaves were collected at different time points throughout 24h light/dark period (Time points are same as mentioned in Fig 1) showing changes in the levels of metabolites during circadian cycle.
(TIF)

S1 Table Experimental conditions of previous HR-MAS NMR studies. HR-MAS NMR conditions used in earlier studies for obtaining metabolic profile in various plant materials.
(DOCX)
