# Supplementary figures and images for: Metabolic Profiling of Intact Arabidopsis thaliana Leaves during Circadian Cycle Using 1H High Resolution Magic Angle Spinning NMR
Source: PLoS One. 2016 Sep 23;11(9):e0163258. doi: 10.1371/journal.pone.0163258 (PMC5035067; doi:10.1371/journal.pone.0163258)

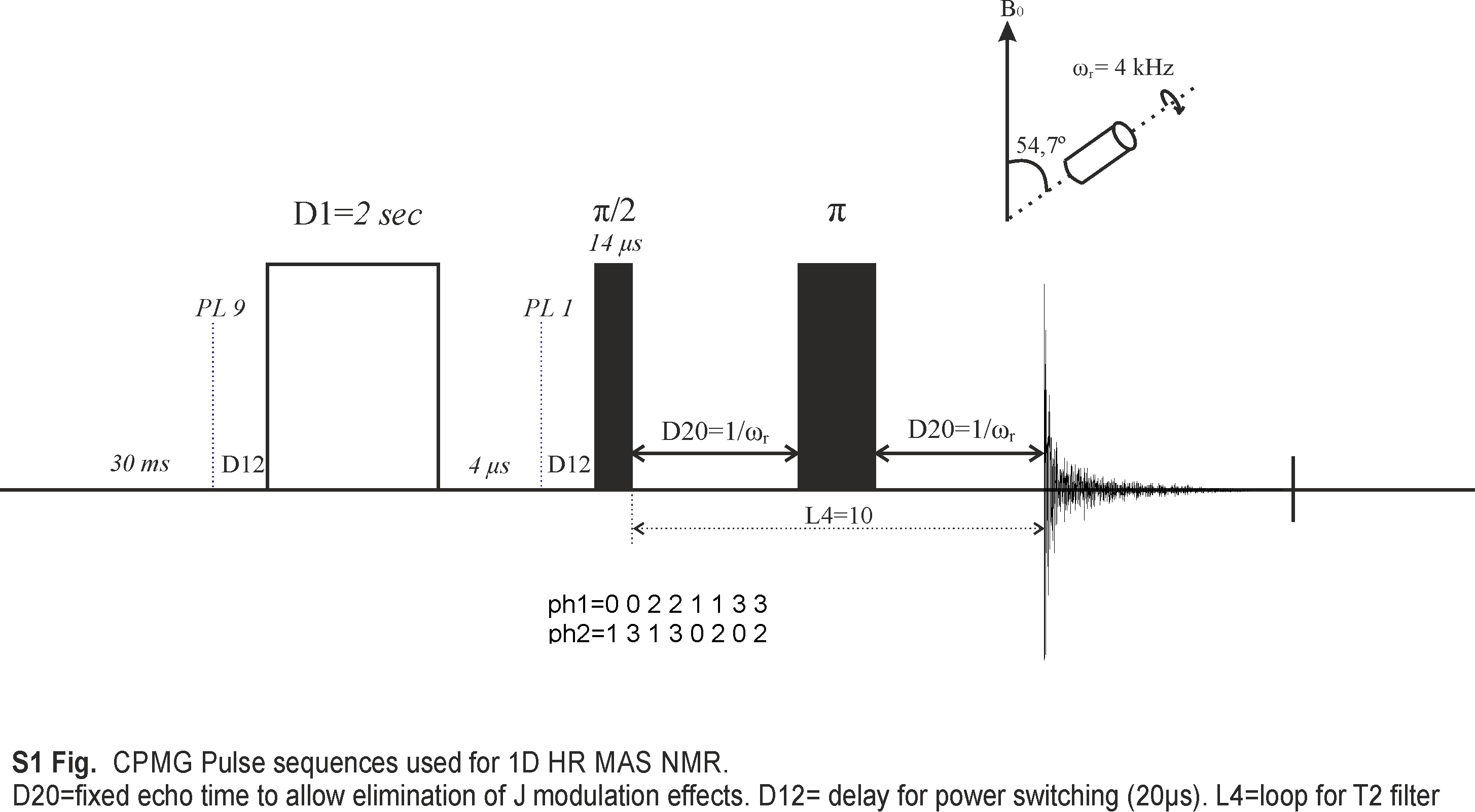

Supplement: S1 Fig — D20 = fixed echo time to allow elimination of J modulation effects. D12 = delay for power switching (20μs). L4 = loop for T2 filter. (TIF) [file pone.0163258.s001.tif]

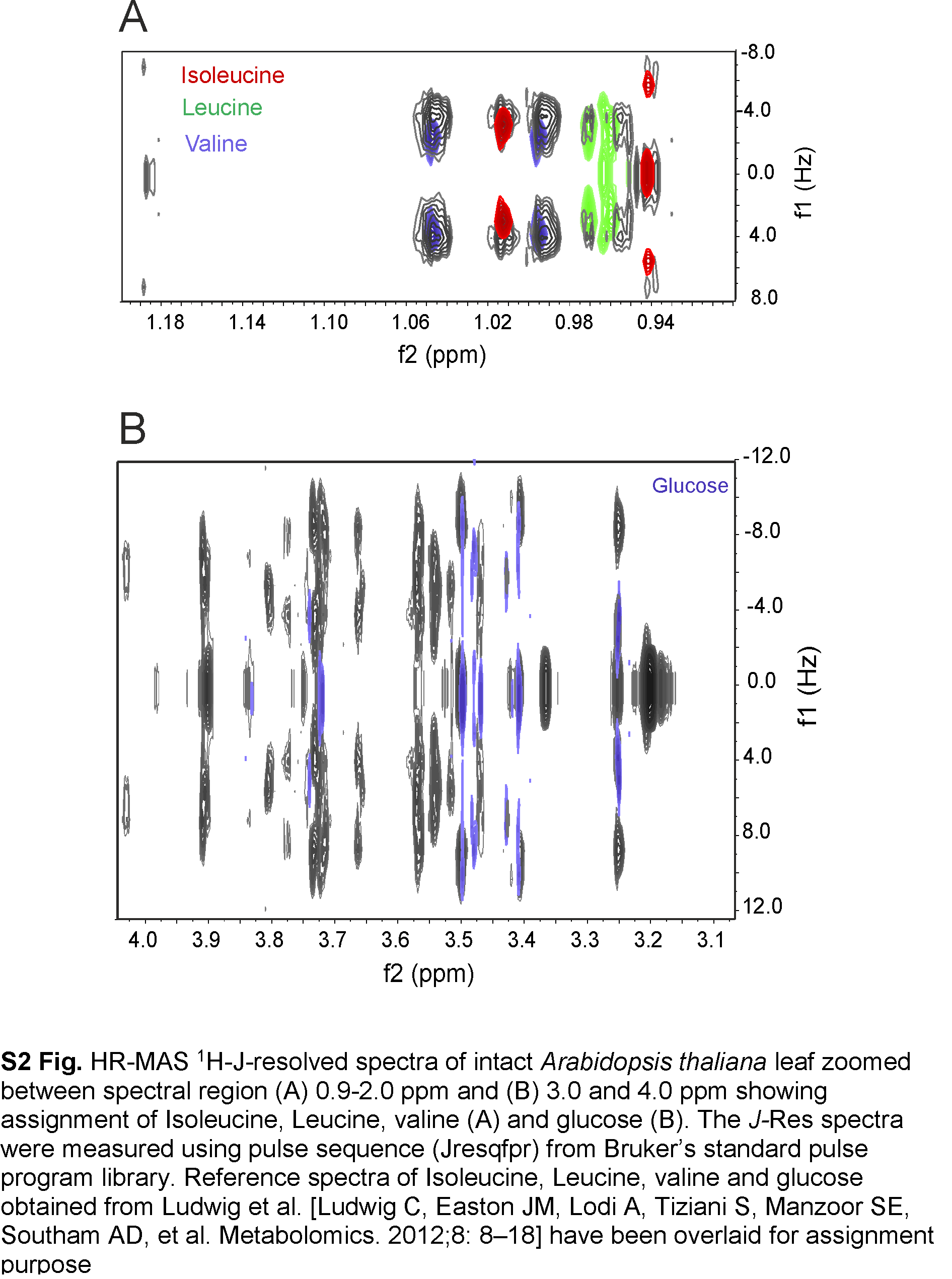

Supplement: S2 Fig — HR-MAS 1H-J-resolved spectra of intact Arabidopsis thaliana leaf zoomed between spectral region (A) 0.9–2.0 ppm and (B) 3.0 and 4.0 ppm showing assignment of Isoleucine, Leucine, valine (A) and glucose (B). The J-Res spectra were measured using pulse sequence (Jresqfpr) from Bruker’s standard pulse program library. Reference spectra of Isoleucine, Leucine, valine and glucose obtained from Ludwig et al. [Ludwig C, Easton JM, Lodi A, Tiziani S, Manzoor SE, Southam AD, et al. Metabolomics. 2012;8: 8–18] have been overlaid for assignment purpose. (TIF) [file pone.0163258.s002.tif]

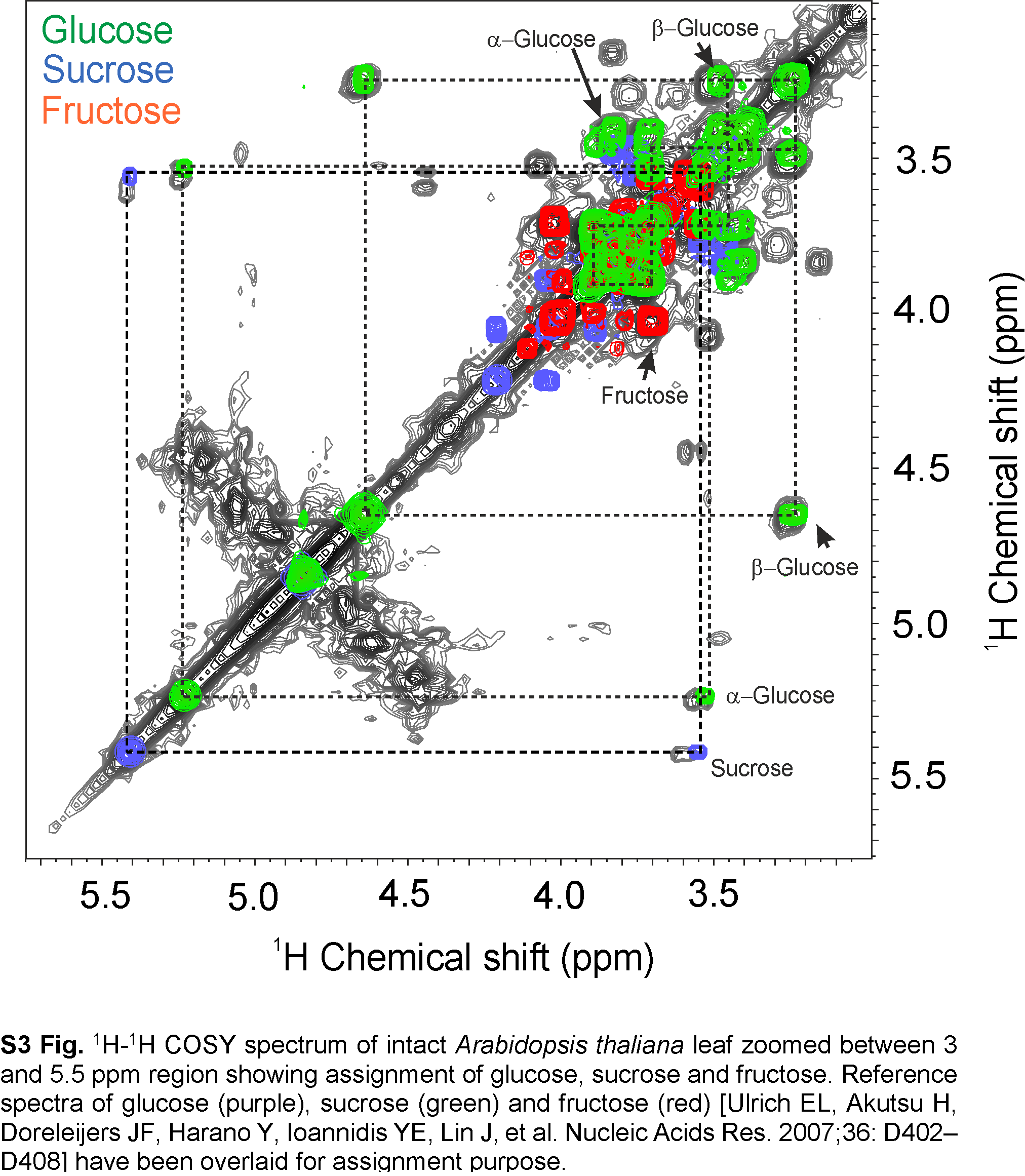

Supplement: S3 Fig — Reference spectra of glucose (purple), sucrose (green) and fructose (red) [Ulrich EL, Akutsu H, Doreleijers JF, Harano Y, Ioannidis YE, Lin J, et al. Nucleic Acids Res. 2007;36: D402–D408] have been overlaid for assignment purpose. (TIF) [file pone.0163258.s003.tif]

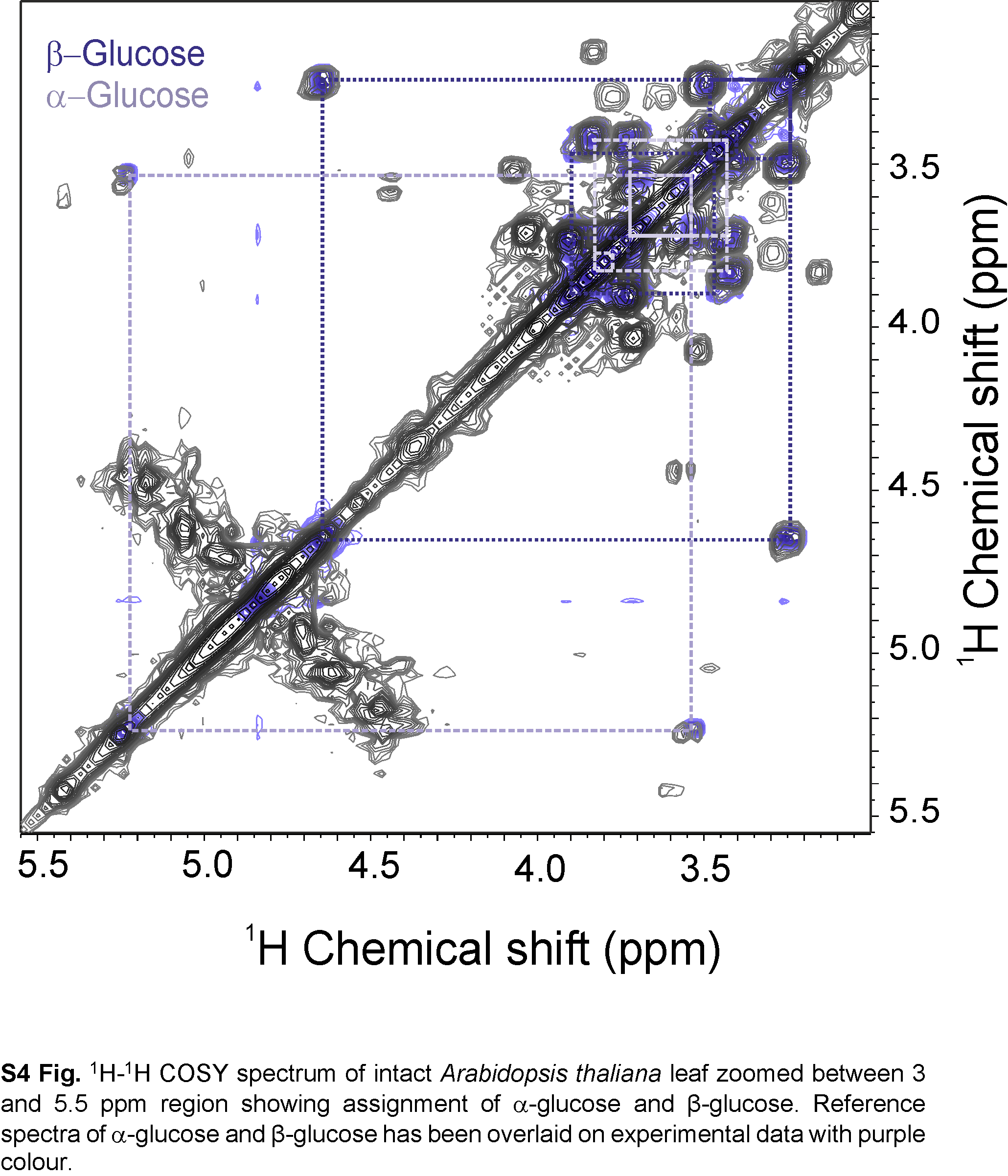

Supplement: S4 Fig — Reference spectra of α-glucose and β-glucose has been overlaid on experimental data with purple colour. (TIF) [file pone.0163258.s004.tif]

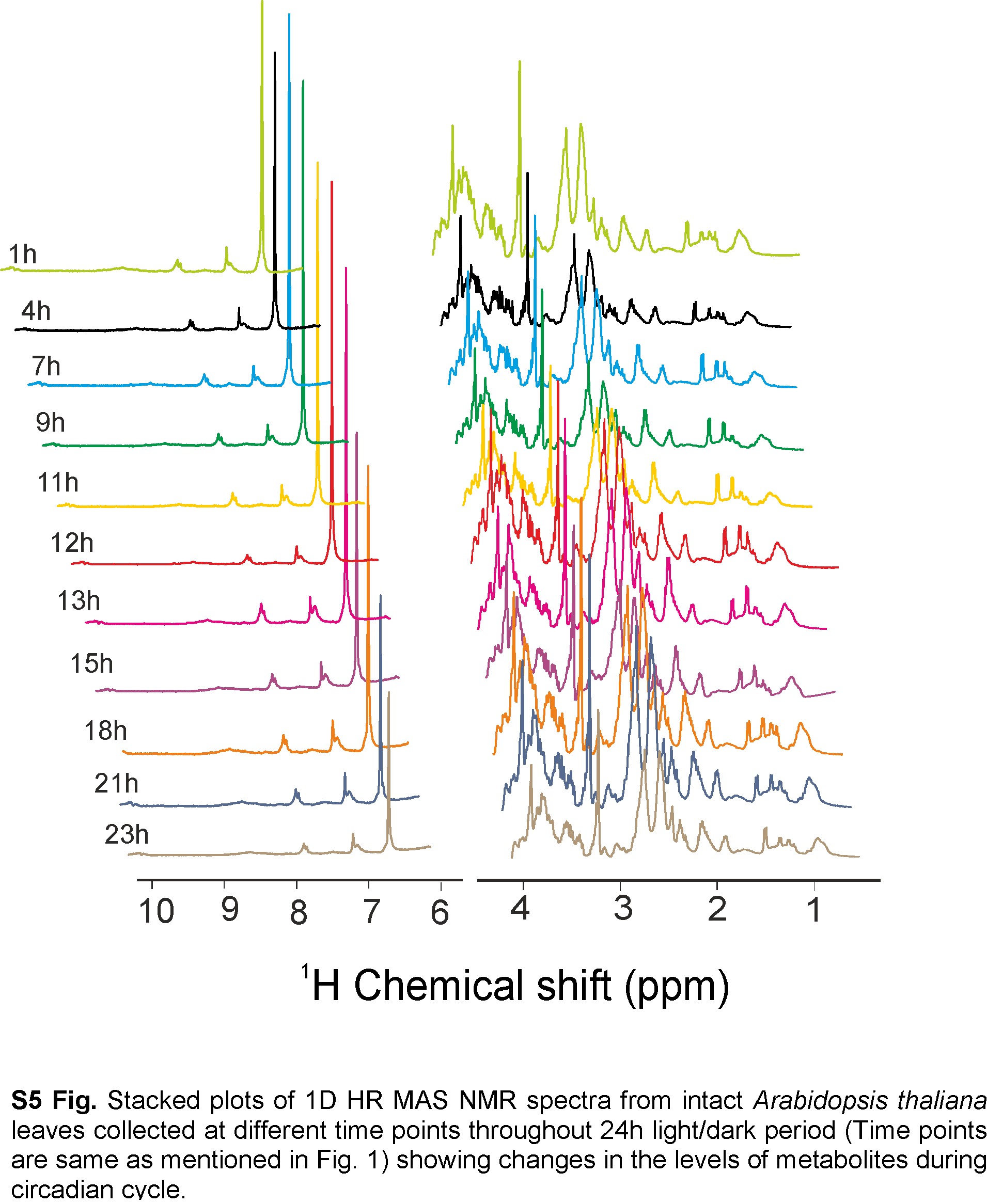

Supplement: S5 Fig — Leaves were collected at different time points throughout 24h light/dark period (Time points are same as mentioned in Fig 1) showing changes in the levels of metabolites during circadian cycle. (TIF) [file pone.0163258.s005.tif]
