# Supplementary material for: Metabolic Profiling of Intact Arabidopsis thaliana Leaves during Circadian Cycle Using 1H High Resolution Magic Angle Spinning NMR
Source: PLoS One. 2016 Sep 23;11(9):e0163258. doi: 10.1371/journal.pone.0163258 (PMC5035067; doi:10.1371/journal.pone.0163258)
Supplement: S1 Table — HR-MAS NMR conditions used in earlier studies for obtaining metabolic profile in various plant materials. (DOCX) [file pone.0163258.s006.docx]

**Supplementary Table**

**S1 Table:** Experimental conditions of previous HR-MAS NMR studies for obtaining metabolic profile in various plant materials

| **References** | **Plant** | **Magnetic field strenght (MHz)** | **Spinning speed (kHz)** | **Temp (K)** | **Pulse sequences** | **Other information** |
| --- | --- | --- | --- | --- | --- | --- |
| Bharti et al. (2011) [15] | *Withania somnifera* | 800 | 8 | 293 (20C) | NOESY, CPMG-pr, COSY, HSQC | Line broadening = 0.3 Hz |
| Sidhu et al. (2010) [16] | *Jatropha curcas* | 400 | 4 | 298 (25C) | NOESY, CPMG-pr, COSY |  |
| Sekiyama et al. (2010) [17] | *Arabidopsis thaliana* | 500 | 8 | 298 | HSQC | Extraction from the leaves |
| Ritota et al. (2010) [18] | *Capsicum annum* | 400 | 7 | 298 | NOESY, zgpg30, TOCSY, HSQC |  |
| Ritota et al. (2012) [19] | *Allium sativum* | 400 | 7 | 298 | NOESY, zgpg30, TOCSY, HSQC | Line broadening = 0.3 Hz |
| De Oliveira et al. (2014) [20] | Swingle’ citrumelo (*Citrus paradisi*  *× Poncirus trifoliata)* | 500 | 5 | 301 (28) | composite pulse presaturation, HSQC, TOCSY | Line broadening = 0.3 Hz |
| Mori et al. (2015) [21] | *Popler hybrid (Populus tremula L.×Populus tremuloides Michx)* | 400 and 500 | 4, 6 and 10 | 298 | HSQC |  |
| Komatsu et al. (2015) [22] | *Euglena gracilis* | 500 | 5 |  | HSQC |  |
